# Nutritional Risks of Heavy Metals in the Human Diet—Multi-Elemental Analysis of Energy Drinks

**DOI:** 10.3390/nu16244306

**Published:** 2024-12-13

**Authors:** Katarzyna Czarnek, Małgorzata Tatarczak-Michalewska, Grzegorz Wójcik, Agnieszka Szopa, Dariusz Majerek, Karolina Fila, Muhammed Hamitoglu, Marek Gogacz, Eliza Blicharska

**Affiliations:** 1 Department of Basic Medical Sciences, Faculty of Medical, The John Paul II Catholic University of Lublin, Konstantynów 1 H St., 20-708 Lublin, Poland; 2 Department of Pathobiochemistry and Interdisciplinary Applications of Ion Chromatography, Medical University of Lublin, 1 Chodźki St., 20-093 Lublin, Poland; malgorzatatatarczakmichalewska@umlub.pl; 3 Department of Inorganic Chemistry, Institute of Chemical Sciences, Faculty of Chemistry, Maria Curie-Skłodowska University, Maria Curie-Skłodowska Sq. 2, 20-031 Lublin, Poland; grzegorz.wojcik2@mail.umcs.pl; 4 Department of Medicinal Plant and Mushroom Biotechnology, Faculty of Pharmacy, Jagiellonian University, 9 Medyczna St., 30-688 Kraków, Poland; a.szopa@uj.edu.pl; 5 Department of Applied Mathematics, Faculty of Mathematics and Information Technology, Lublin University of Technology, Nadbystrzycka 38 St., 20-618 Lublin, Poland; d.majerek@pollub.pl; 6 Department of Chemistry, Faculty of Food Science and Biotechnology, University of Life Sciences, 15 Akademicka St., 20-950 Lublin, Poland; karolina.fila@up.lublin.pl; 7 Department of Pharmaceutical Toxicology, Faculty of Pharmacy, Yeditepe University, 34755 Istanbul, Turkey; mohammad.saz@yeditepe.edu.tr; 8 2nd Chair and Department of Gynecology, Medical University of Lublin, 20-090 Lublin, Poland; marek.gogacz@umlub.pl

**Keywords:** food safety, public health, health risks, food security, trace elements, heavy metals, energy drinks, mineral composition, ICP-OES, ICP-MS

## Abstract

**Background:** In recent years, the consumption of energy drinks (EDs) by adolescents and young adults has increased significantly, so concerns have been raised about the potential health risks associated with excessive ED consumption. Most analyses on EDs focus on the caffeine content. Research on the content of minerals (essential and toxic) in energy drinks can be considered scarce. Therefore, there is a need for research stating the actual status of heavy metal content in commercially available energy drinks. **Methods:** This research presents the determination of the total concentrations of macro-elements and trace elements (TEs), such as Na, K, Mg, Ca, Al, Cr, Co, Cu, Fe, Mn, Ni, B, Zn, V, Sr, Ba, Pb, Cd, and As in nine samples of energy drinks using inductively coupled plasma optical emission spectrometry (ICP-OES) and inductively coupled plasma mass spectrometry (ICP-MS) techniques. **Results:** The order in the content of macro-minerals in the EDs was as follows: Na > K > Mg > Ca. The results showed that ED 1, ED 3, and ED 7 samples had the highest micro-mineral concentrations. All the samples had a hazard quotient and hazard index < 1, indicating no non-carcinogenic risk from exposure to single or multiple heavy metals in both the adolescent and adult age groups. Some samples exceeded the threshold limit of acceptable cancer risk for As, Ni, and Cr in both adolescents and adults. **Conclusions:** This assessment showed that in addition to health implications based on the caffeine content of EDs, there might be a carcinogenic risk associated with the toxic element content of these beverages. This research also highlights notable differences in the TE levels among various ED brands, which may have important implications for consumer well-being and health.

## 1. Introduction

In recent years, the consumption of energy drinks (EDs) by adolescents and young adults has risen significantly, but concerns have been raised about the potential health risks associated with excessive consumption. Such drinks are aggressively advertised, and are easily accessible in local stores, supermarkets, and gas station stores in almost all European countries [1,2,3,4,5]. EDs are marketed as enhancers of mental acuity and physical performance. Adolescents gravitate towards these beverages to swiftly boost energy levels, and increase scholastic or athletic performance [1]. EDs have a high caffeine content which is normally combined with large amounts of vitamins, minerals, taurine, amino acids, and herbal extracts [6,7,8]. Most EDs contain from 50 to 505 mg of caffeine per can or bottle. Research under controlled conditions determined that 200 mg of caffeine induces atrial flutter and atrial fibrillation [1,9,10]. A growing number of case reports identifying various adverse effects following the acute ingestion of EDs, namely acute hemodynamic perturbations, disturbances in vascular function, and other cardiovascular abnormalities, have been reported [7,11,12,13,14]. Moreover, there have been reports of regular energy drink consumption being associated with anxiety and sleep disturbances [15,16].

Most analyses on EDs focus on the caffeine content. Besides caffeine, EDs generally contain high sugar and other components such as B-complex vitamins, taurine, ginseng, guarana seed extract, yerba mate, acai, and Ginkgo biloba. Some of these components (e.g., plant extracts) may lead to contamination problems in final products, including pesticide and heavy metal (HM) residues [17]. Energy drinks could also be contaminated during processing and packaging. According to some studies, most of the HMs present in alcoholic beverages can originate from agricultural pesticides or contamination from processing equipment (pipelines, containers, tanks, filtration systems, aluminum cans, etc.) [18,19,20].

The term “heavy metal” is based on categorization by density or molar mass. HMs are naturally occurring elements that have high atomic numbers and densities that are five times higher than water [21]. It is often used as a group name for metals (i.e., transition metals from vanadium to zinc) that are associated with contamination and potential toxicity. All so-called “heavy metals” and their compounds may have relatively high toxicity (e.g., lead or cadmium). Lead (Pb) is a toxic metal affecting nearly all organs, mimicking essential elements like calcium, iron, and zinc, and disrupting enzyme systems critical for heme synthesis, cell development, and bone growth. Arsenic (As), a widely distributed toxic metalloid, is found in water, air, food, and soil. It inactivates up to 200 enzymes involved in energy production, DNA synthesis, and repair, disrupting ATP production. Chronic exposure to As causes multisystem dysfunction and is a known carcinogen. Cadmium (Cd), a toxic element with no biological role, accumulates in plants and animal tissues, particularly the liver and kidneys. Prolonged exposure through air, water, and food can lead to cancer and damage to skeletal, urinary, cardiovascular, and nervous systems. Contamination during raw material processing may introduce Cd into beverages [22]. Aluminum (Al), abundant in the earth’s crust, primarily enters the body through food. It disrupts the absorption of fluoride, calcium, and iron, and its phosphorus-binding properties may lead to phosphate depletion and osteomalacia. Al’s main toxic effect is neurotoxicity [23]. Chromium occurs in various forms. Hexavalent chromium (Cr VI) is a Group 1 human carcinogen, while trivalent chromium (Cr III) is essential for carbohydrate, lipid, and protein metabolism and supports normal glucose regulation, with no classified carcinogenicity [24].

Nonetheless, metals are not always toxic and some are in fact essential and, depending on the dose and exposure levels and the receiving organism/population, the balance between essential or toxic may tip (e.g., iron or zinc) [25,26]. Trace elements (TEs) are classified as major minerals (macro-minerals) and trace minerals (micro-minerals). Major minerals include several elements such as Ca, Na, Mg, and K; while trace minerals include Zn, Cu, Fe, Mn, Co, B, and Cr. About 20 of the known elements are qualified today as essential [27]. Many TEs are essential to numerous biological, chemical, and molecular processes, regulating cellular homeostasis, humoral, and cellular immune responses, and being cofactors of many enzymes and antioxidant molecules [28,29]. Some metals, such as Fe, Zn, Cu, Co, and Mn, are required for various physiological functions in humans at low concentrations, but they become toxic at higher concentrations. Other HMs, such as cadmium and lead, are not known to have any beneficial effects on human health and their accumulation in the human body is deleterious to health [30].

The available research on the mineral content (both essential and toxic) in energy drinks remains limited. This study aimed to quantify the concentrations of Na, K, Mg, Ca, Al, Cr, Co, Cu, Fe, Mn, Ni, B, Zn, V, Sr, Ba, Pb, Cd, and As in nine commercially available energy drinks sold in Poland, packaged in aluminum cans. Inductively coupled plasma optical emission spectroscopy (ICP-OES) and inductively coupled plasma mass spectrometry (ICP-MS) were employed for the elemental analysis. Additionally, a toxicological risk assessment was conducted to evaluate the potential health risks associated with exposure to these elements through energy drink consumption in adolescents and adults. To our knowledge, this is the only study that estimated the carcinogenic risk for toxic elements from the consumption of EDs.

## 2. Materials and Methods

### 2.1. Samples and Sample Preparation

Nine different brands of energy drink samples were obtained from markets in Lublin, Poland. The energy drinks selected for research represent the most popular and most widely consumed brands in Poland based on market data. All of the packaging materials of the energy drink samples analyzed in the study were aluminum cans. The composition of each ED is shown below in Table 1.

To eliminate any dissolved CO_2_, the samples were sonicated using an ultrasonic bath for 70 min. In the next step, 9 mL of the degassed sample was transferred to a Teflon cuvette and 3 mL of 65% (*v*/*v*) HNO_3_ (Suprapur, Merck, Darmstadt, Germany) was added. The components were thoroughly mixed. The reaction vessel was closed and placed in a microwave mineralizer Multiwave 5000 (Anton Paar, Graz, Austria). The program used was based on temperature control with the dynamic selection of microwave power: temperature was increased from ambient temperature to 180 °C in 20 min., held at a temperature of 180 °C for 10 min., and cooled to 70 °C.

After the cooling to ambient temperature, the digested samples were diluted to 25 mL with ultrapure water, i.e., deionized water of resistance 18.3 MΩ cm^−1^ (EASYpure™ system, Barnstead, Thermolyne Corporation, Ramsey, MN, USA). The standard solution of elements was prepared by the dilution of ICP multi-element standard solution XXV for MS (Merck, Darmstadt, Germany). All the analyses were performed in triplicate. The analysis of blank samples confirmed the high purity of the used chemicals and containers.

### 2.2. ICP-OES Conditions

The ICP-OES analysis was performed by using a Varian 720-ES spectrometer (Varian, Melbourne, Australia). The content of four macro-minerals Na, K, Mg, and Ca was determined by this method (Table 2).

### 2.3. ICP-MS Conditions

The ICP-MS analysis was performed by using an Agilent Technologies 7700x series spectrometer (Agilent, Tokyo, Japan). The content of the fifteen elements B, Al, V, Cr, Mn, Fe, Co, Ni, Cu, Zn, As, Sr, Cd, Ba, and Pb was determined by this method (Table 3).

### 2.4. Toxicological Risk Assessment

The toxicological risk assessment for the investigated elements in the ED samples was conducted using the established equations to estimate the estimated daily intake (EDI) and non-carcinogenic risks (HQ) [22,31,32].

The EDI was calculated using the following equation:

EDI = (C × IR × EF × EXD)/(BW × AT)

The hazard quotient (HQ) for each element was calculated as follows:

HQ = EDI/RfD

where EDI is the estimated daily intake (mg/kg/day); C is the metal concentration in samples (mg/L); IR is the daily intake rate of EDs at 0.23 L/day for adolescents and 0.16 L/day for adults; EF is exposure frequency, in this study, 240 day/year; EXD is the exposure duration, in this study, 8 years (10–18 years old) for adolescents and 47 years (18–65 years old) for adults [33]; BW is the body weight at 45 kg for adolescents and 70 kg for adults; AT = EXD × 365 days is the averaging time for non-carcinogensis; HQ is the hazard quotient; and RfD is the reference oral dose in mg/kg/day (0.2 for B, 1 for Al, 0.009 for V, 0.003 for Cr, 0.14 for Mn, 0.7 for Fe, 0.0003 for Co, 0.02 for Ni, 0.04 for Cu, 0.3 for Zn, 0.0003 for As, 0.6 for Sr, 0.0005 for Cd, 0.07 for Ba and 0.0035 for Pb).

The average daily intake in our study, based on the EFSA report, was approximately 0.23 and 0.16 L/day for adolescents and adults, respectively. Adolescents (10–18 years) consumed the most, averaging 7 L per month, at least 4–5 times a week. Adults (18–65 years) averaged 4.5 L monthly, with at least 4–5 times a week [33].

To account for the additive effects of exposure to multiple elements, the total hazard index (HI) was calculated as the arithmetic sum of individual HQ values:

HI=∑HQ

The incremental lifetime cancer risk (ILCR) was estimated to determine the probability of developing cancer over a lifetime due to exposure to potential carcinogenic elements. The formula used was as follows:

ILCR = EDI × CSF

where CSF is the cancer slope factor (mg/kg/day). The oral values used were 0.5 for Cr, 1.7 for Ni, 1.5 for inorganic As, 0.38 for Cd, and 0.0085 for Pb. The CSF is a toxicity value that quantitatively defines the relationship between dose and response. An ILCR value greater than 10^−4^ indicates high cancer risk, while an ILCR value smaller than 10^−6^ is acceptable and safe, and a value in the range of 10^−6^ to 10^−4^ indicates a moderate risk [22,34].

### 2.5. Statistical Analysis

To analyze element concentrations in various energy drink products, the Kruskal–Wallis H test was used, which is a non-parametric method suitable for non-normally distributed data often seen in environmental studies. Each product was sampled three times (*n* = 3), and the test compared multiple independent groups to detect significant differences in element levels [35,36]. To control the family-wise error rate, Bonferroni adjustments were applied to the *p*-values, reducing the risk of Type I errors and ensuring robust significance levels despite the small sample size [37].

One-sample Student’s *t*-tests were conducted to determine if element concentrations in individual drinks exceeded the normative values set by bodies like WHO (World Health Organization), EU (European Union), and US EPA (United States Environmental Protection Agency). Mean concentrations from three samples per product were compared to these norms. Despite the limited sample size (*n* = 3), the one-sample *t*-test was deemed appropriate for detecting significant exceedances. Bonferroni adjustments were also applied to control for multiple hypothesis testing, ensuring the results’ reliability [38].

All the analyses were performed in R (version 4.4.1) using the tidyverse suite for data manipulation and visualization, and the rstatix package for statistical tests and Bonferroni corrections [39,40,41]. This approach ensured rigor, reproducibility, and adherence to statistical best practices.

## 3. Results

Nine energy drinks of different brands were analyzed using the techniques ICP-OES and ICP-MS, and the results are presented in Table 4 and Table 5. The total concentrations of 19 elements (Na, K, Mg, Ca, B, Al, V, Cr, Mn, Fe, Co, Ni, Cu, Zn, As, Sr, Cd, Ba, and Pb), both essential and toxic, were determined in the ED samples.

### 3.1. Macro-Mineral Composition Analysis of Energy Drinks

Using the ICP-OES method, the content of four macro-minerals (Na, K, Mg, and Ca) was determined, the concentrations of which, summarized in Table 4, are given in mg L^−1^.

Among the measured elements, sodium was the element present in the investigated products at the highest concentration. The content of this macro-mineral ranged from 8.62 mg L^−1^ (ED 9) to 619.85 mg L^−1^ (ED 4). Potassium concentrations in the products tested were generally lower than Na concentrations, ranging from 5.51 mg L^−1^ (ED 3) to 189.57 mg L^−1^ (ED 5).

The determined calcium concentrations ranged from 2.54 mg L^−1^ (ED 2) to 116.1 mg L^−1^ (ED 3). The magnesium content ranged from 0.013 mg L^−1^ (ED 2) to 196 mg L^−1^ (ED 3), and was significantly higher (543.97 mg L^−1^–ED 9) for only one ED enriched with this element at the production stage.

The Kruskal–Wallis test showed significant differences in the concentrations of all the analyzed macro-minerals—Ca, K, Mg, and Na—between the different brands of energy drinks. For each of these elements, the *p*-values after Bonferroni correction (*p.adj*) were significantly less than 0.05 (Ca: 0.00692, K: 0.00552, Mg: 0.00476, and Na: 0.00524), which indicates that there are significant differences in their concentrations between the products tested. The number of samples for each element was 27, and the test statistics exceeded the critical values for degrees of freedom of 8, which confirms the significance of the observed differences.

### 3.2. Micro-Mineral Composition Analysis of Energy Drinks

**Figure 1:**
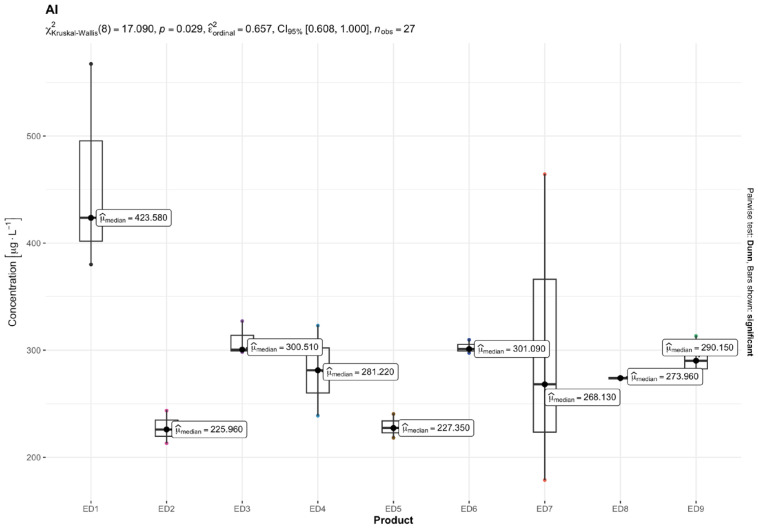
Content of aluminum (Al) [mg L^−1^] in the analyzed energy drinks (*n* = 3).

**Figure 2:**
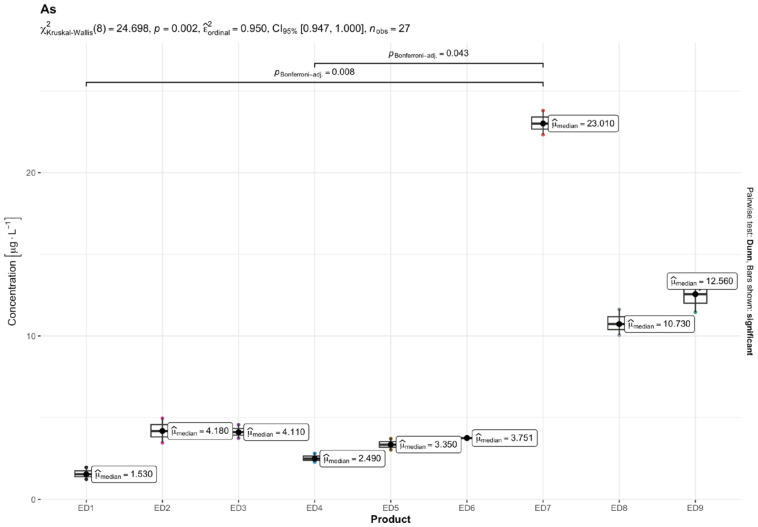
Content of arsenic (As) [mg L^−1^] in the analyzed energy drinks (*n* = 3).

**Figure 3:**
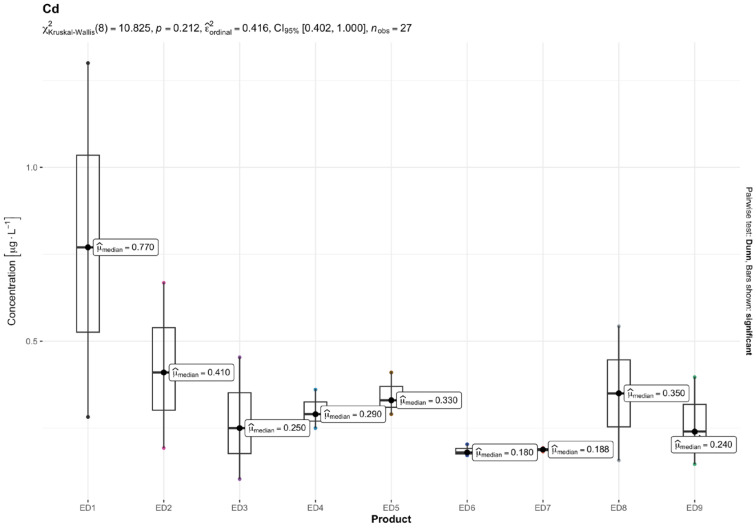
Content of cadmium (Cd) [mg L^−1^] in the analyzed energy drinks (*n* = 3).

**Figure 4:**
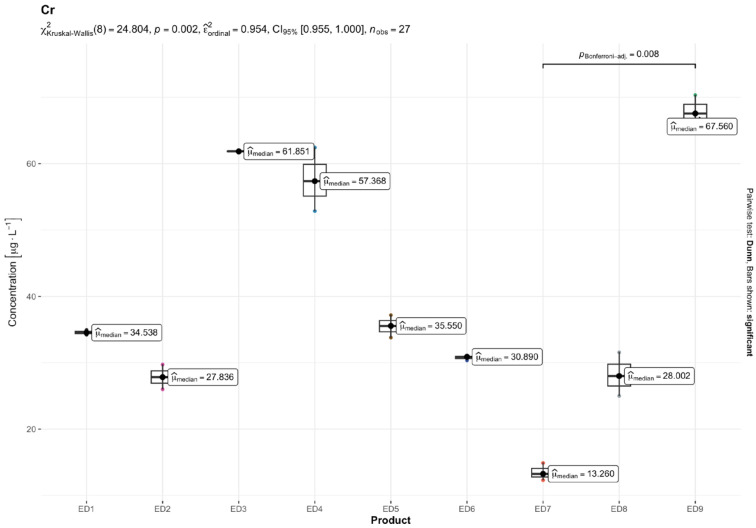
Content of chromium (Cr) [mg L^−1^] in the analyzed energy drinks (*n* = 3).

**Figure 5:**
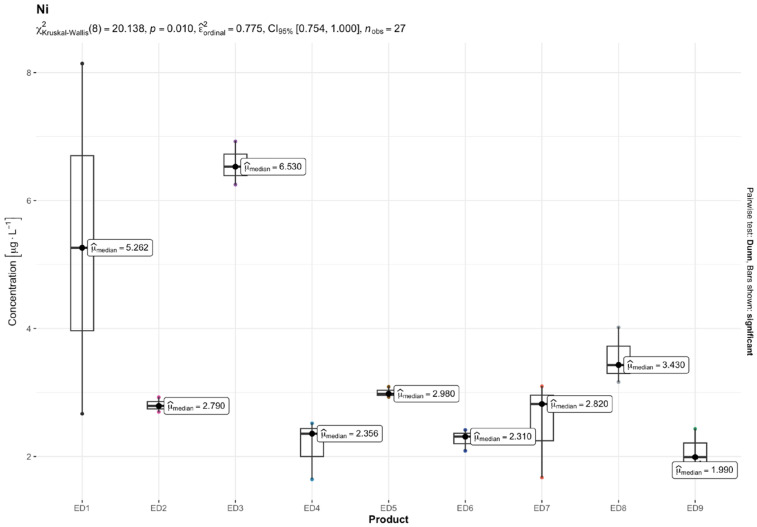
Content of nickel (Ni) [mg L^−1^] in the analyzed energy drinks (*n* = 3).

**Figure 6:**
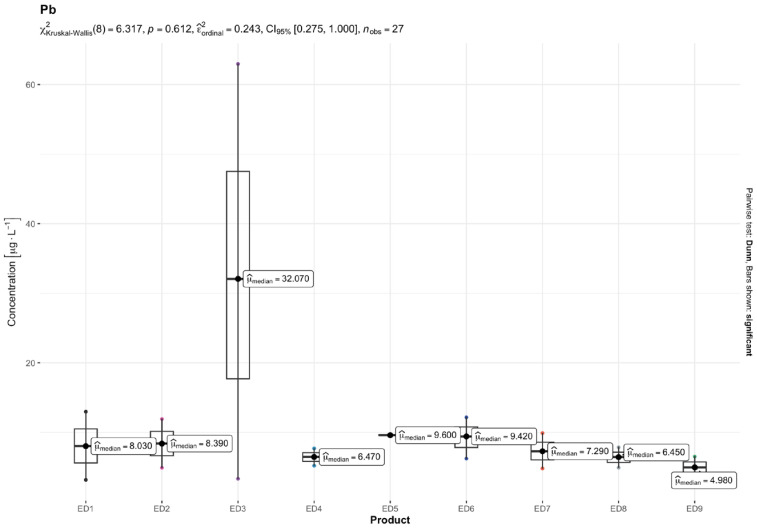
Content of lead (Pb) [mg L^−1^] in the analyzed energy drinks (*n* = 3).

The results of quantitative determinations of fifteen micro-minerals (B, Al, V, Cr, Mn, Fe, Co, Ni, Cu, Zn, As, Sr, Cd, Ba, and Pb), obtained using the ICP-MS technique, are given in µg L^−1^ of the initial drink solution in Table 5 and in Figure 1, Figure 2, Figure 3, Figure 4, Figure 5 and Figure 6.

Of the elements listed above, the essential TEs include Fe, B, Cu, Cr, Mn, Zn, and Co, with Fe, Cu, Cr, and Zn also classified as heavy metals. Conversely, Ni, As, Cd, and Pb are examples of HMs that do not have any biological role. Some trace elements like Al, V, Sr, and Ba have not been demonstrated to be essential in humans.

Among the essential TEs analyzed, B and Fe exhibited the greatest levels in the examined EDs. The B concentration varied between 194.01 µg L^−1^ in ED 3 and 796.82 µg L^−1^ in ED 7, with the Fe levels ranging from 121.86 µg L^−1^ in ED 2 to 308.31 µg L^−1^ in ED 7. Concentrations for the other essential TEs varied within the following ranges: Mn (6.69–107.27 µg L^−1^), Cr (13.49–67.53 µg L^−1^), Zn (10.34–64.56 µg L^−1^), Cu (2.94–16.76 µg L^−1^), and Co (0.19–4.13 µg L^−1^).

Among the most hazardous toxic elements, Pb and As exhibited the highest concentration in th EDs. The average Pb levels in the energy drinks tested were consistent (ranging from 5.00 to 9.61 µg L^−1^), except for one ED 3 sample, which contained 32.79 µg L^−1^ of Pb. The highest concentrations of Ni and As were found in ED 3 and ED 7, with concentration ranges of 2.04–6.57 µg L^−1^ and 1.57–23.05 µg L^−1^, respectively. Cd was found in very small amounts in the EDs tested, ranging from 0.19 µg L^−1^ in ED 7 and ED 6 to 0.78 µg L^−1^ in the ED 1 sample.

It was noticed that Al was present in the highest concentrations among the non-essential TEs, with a range from 227.54 µg L^−1^ (ED 2) to 456.97 µg L^−1^ (ED 1). Toxic Ba was also found in the ED samples (the lowest concentration of 4.81 µg L^−1^ was recorded for ED 7, and the highest 19.98 µg L^−1^ for ED 3). Interestingly, of the EDs tested, the highest Sr concentration was reported for the ED 3 sample, which was 3878.21 µg L^−1^. Unlike the sample of ED 3, the other EDs had low Sr concentrations (between 2.99 and 72.36 µg L^−1^). Additional non-essential TEs, like V, were found in the EDs analyzed at levels between 0.29 (ED 9) and 10.44 µg L^−1^ (ED 1).

The result of the Kruskal–Wallis test for micro-minerals indicates that for some elements, there are significant differences in concentrations between product groups, while for others, these differences are not statistically significant after taking into account the Bonferroni correction. Trace elements such as As, Ba, Co, Cr, Cu, Sr, V, and Zn showed significant differences in concentrations between products. The values of the adjusted *p* (*p.adj*) for these trace elements were lower than the accepted level of significance (e.g., 0.05), which suggests that their concentrations differ depending on the type of product. On the other hand, trace elements such as Al, B, Cd, Fe, Mn, Ni, and Pb did not show significant differences after applying the Bonferroni correction. This means that the differences in their concentrations between the tested product groups are not statistically significant, so it cannot be stated that these products differ significantly in terms of their content. In summary, the Kruskal–Wallis test showed significant differences in the concentrations of some micro-minerals, which indicates that some of them may be more dependent on the type of product. The Bonferroni correction provided control for the first-type error in the multiple comparison analysis, allowing a more conservative assessment of statistical significance.

The EDI for fifteen micro-minerals via the consumption of the EDs is summarized in Table 6 and Table 7. The HQ was calculated to evaluate the risk of chronic systemic toxicity posed by exposure to each toxic and essential element. The HQ represents the ratio of EDI to the RfD, which is the maximum acceptable dose assumed to be without adverse health effects in humans. When the HQ value for an element exceeds 1 (HQ > 1), it indicates a potential health risk, meaning the exposure surpasses the maximum permissible RfD. Conversely, if HQ ≤ 1, adverse health effects are unlikely, and the exposed population is presumed to be safe.

The HQ values for the investigated elements through the consumption of the EDs are presented in Table 6 and Table 7. For the nine analyzed ED samples, all the HQ values for both adolescent and adult age groups were below the risk threshold (HQ < 1), indicating no significant non-carcinogenic risks to consumers.

The potential cumulative hazard from exposure to the fifteen micro-minerals was also assessed, with the results presented in Table 6 and Table 7. The HI ranged from 0.10 to 0.32 for the nine ED samples in adolescents and from 0.04 to 0.13 in adults. All the HI values were below the critical threshold of one, confirming that the cumulative exposure to these micro-minerals does not pose a significant non-carcinogenic risk for consumers.

The carcinogenic risk for Cr, Ni, As, Cd, and Pb via the consumption of the EDs for both adolescents and adults was determined and is presented in Table 8. To our knowledge, this is the only study that estimated the carcinogenic risk for toxic elements from the consumption of EDs. For Pb and Cd, the ILCR values were consistently below the safe limit of 10^−6^ for both adolescents and adults, indicating no carcinogenic concern for these metals.

For As, in adolescents, the ILCR values for most samples (except ED 1 and ED 7) ranged between 10^−6^ and 10^−4^, signifying a moderate cancer risk. ED 1 was within the safe limit, while ED 7 exceeded the 10^−4^ threshold, indicating a high cancer risk for this age group. Among adults, the ILCR values for As ranged from 3.3 × 10^−6^ (ED 1) to 4.9 × 10^−5^ (ED 7). While most samples were below the safe limit, ED 7, ED 8, and ED 9 indicated a moderate risk (10^−6^ to 10^−4^).

For Ni, the ILCR values in adolescents ranged from 1.2 × 10^−5^ (ED 9) to 4.7 × 10^−5^ (ED 8), all within the moderate risk range of 10^−6^ to 10^−4^. For adults, most samples fell within the safe limit (<10^−6^), except ED 1 (1.3 × 10^−5^) and ED 3 (1.6 × 10^−5^), which presented a moderate carcinogenic risk.

For Cr, the ILCR values in adolescents ranged from 2.4 × 10^−5^ (ED 7) to 1.1 × 10^−4^ (ED 9). Two samples (ED 3 and ED 9) approached or slightly exceeded the 10^−4^ threshold, indicating a high cancer risk, while the others fell within the moderate risk range. In adults, the ILCR values for Cr ranged from 9.9 × 10^−6^ (ED 7) to 4.8 × 10^−5^ (ED 9), with all the samples falling within the moderate risk range.

## 4. Discussion

### 4.1. Energy Drink Macro-Mineral Composition

The results of the energy drinks showed a high content of Na, followed by K, Mg, and Ca. In a previous study, Leśniewicz et al. [4] reported that the concentration of Na in energy drinks consumed in Poland varied from 174 mg L^−1^ to 943 mg L^−1^. In the study by Martins et al. [49], the Na concentrations in the seventeen energy drinks were very different depending on the type of packaging; they ranged from 31.5 to 1216.7 mg L^−1^ in PET bottles and 8.73 to 630.4 mg L^−1^ in aluminum cans. The results obtained in our study (8.62 mg L^−1^–619.85 mg L^−1^) are similar to them. The potassium concentrations determined by Martins’ research team ranged from 2.4 to 210 mg L^−1^ [49]. The results of our experiments are consistent with this range. The relationship between sodium and potassium at the cellular level is responsible for many essential functions, including maintaining fluid balance. Na and K play important roles in various bodily functions, and an imbalance in their intake can have significant health implications, particularly concerning cardiovascular diseases and hypertension. Reducing the amount of Na intake in foods remains an essential concern for the food processing industry. The WHO recommends a Na–K molar ratio of <1 to help lower blood pressure. Therefore, it is very important to monitor the content of these elements in energy drinks, which are becoming an important part of the diet of young people [50,51,52,53,54].

The Mg and Ca levels in our study ranged from 0.013 mg L^−1^ to 196 mg L^−1^ and 2.54 mg L^−1^ to 116.1 mg L^−1^, respectively. Of all the macro-minerals tested, the largest differences between the minimum and maximum concentrations were obtained for Mg, as have other researchers. Calcium concentrations determined by the ICP-OES technique by other researchers ranged from 12.9 mg L^−1^ to 106 mg L^−1^ [49], and from 2.39 mg L^−1^ to 120 mg L^−1^ [55], so they were consistent with our results. In contrast, the Ca levels determined by Leśniewicz et al. [4] and Mohammed et al. [56] were lower, not exceeding 41.2 mg L^−1^ and 66 mg L^−1^, respectively.

### 4.2. Energy Drink Micro-Mineral Composition

The concentrations of V, B, and Co in EDs were examined only in the study conducted by Leśniewicz et al. [4], with the levels ranging from 0.19 to 4.25 μg L^−1^, 36.3 to 92.8 μg L^−1^, and 0.41 to 1.63 μg L^−1^, respectively. These findings showed a considerable decrease in comparison to our research, where the highest average levels of these substances were 10.44 μg L^−1^ (for V), 796.82 μg L^−1^ (for B), and 4.13 μg L^−1^ (for Co). The WHO guidelines for drinking water quality do not establish a specific limit for Co, V, and Sr, among others [42]. We found that the concentrations of V and Co in our study were within the ranges recommended by the California Office of Environmental Health Hazard Assessment (OEHHA) and US EPA [44,47], respectively (Table 5). B’s outcome was significantly lower than the standard suggested by the WHO. Leśniewicz et al. [4] reported Sr concentrations ranging from 12.8 to 117 μg L^−1^, while Szymczycha-Madeja et al. [55] found concentrations ranging from 1.8 to 608 μg L^−1^. However, our study showed a significant increase in the Sr levels for the ED 3 sample to a value equal to 3878.21 μg L^−1^ (statistic = 918.030) with an adjusted *p*-value of 0.000, indicating that the Sr concentration in this product significantly exceeds the US EPA standards. This supports the hypothesis that the Sr levels in ED 3 are higher than the normative values. The researchers also found Ba levels ranging from 4.28 to 23.7 μg L^−1^ [4] and from 19 to 82 μg L^−1^ [55]. In our research, the levels of Ba found fell within the lowest range reported in their study (4.81–19.98 μg L^−1^).

The findings revealed that in the several ED samples in our research, the Al concentrations showed significant deviations from the EU and US EPA norms (Table 5). Specifically, ED 6 (statistic = 28.372, *p.adj* = 0.006), ED 3 (statistic = 11.656, *p.adj* = 0.033), and ED 8 (statistic = 96.162, *p.adj* = 0.000) all exhibited Al levels that significantly exceed the standards mentioned. The elevated Al levels in the tested EDs are linked to the specific packaging material used. Martins and colleagues [49] found higher levels of aluminum (150–7150 μg L^−1^) in EDs within aluminum cans compared to the levels in PET bottles (10–120 μg L^−1^). Leśniewicz et al. [4] and Bunu et al. [57] found that the highest levels in EDs were 1260 μg L^−1^ and 2049 μg L^−1^, respectively. It is well known that aluminum cans are covered with a thin polymer layer, which may impact the Al content in EDs [49]. Francisco et al. [58] stated that if cans are mishandled, it may result in the beverage coming into contact with the metal can material. Even though the highest average concentration of Al in our study was significantly lower (456.97 μg L^−1^) compared to previous studies by Leśniewicz et al. [4], Martins et al. [49], and Bunu et al. [57], the levels of this metal found still exceed the permissible limits in drinking water as per the specified standards (Table 5). Furthermore, as stated by Kilic et al. [17], Cr, Ni, and Fe are among the other metals that have the ability to move from the metal container into the ED sample. The Al levels in non-carbonated samples ranged from 26.12 μg L^−1^ to 48.16 μg L^−1^, with a mean of 37.74 μg L^−1^, according to Ahmed et al. [59]. The aforementioned study also discovered that orange juice samples had an average Al content of 36.98 μg L^−1^. Fruit juices and non-alcoholic beverage samples were the subject of another study conducted in the USA; the results showed a broad range of concentrations, ranging from 0.017 to 2.6 mg L^−1^, with an average of 0.36 mg L^−1^ [60]. Across different brands in Egypt, the Al content of soft drink samples, whether in glass, plastic, or can bottles, varied from 20 μg L^−1^ to 507.35 μg L^−1^ [61].

Despite these elevated Al levels, the calculated HQs for aluminum in all the tested EDs for adolescents and adults were below the threshold of 1 (HQ ≤ 1), indicating no significant non-carcinogenic risk to consumers. This finding aligns with evidence suggesting that while aluminum is present in various foods, beverages, and water, orally ingested Al at typical levels is not immediately harmful to humans. However, potential long-term exposure risks, including its speculated role in neurodegenerative conditions such as Alzheimer’s disease, remain a topic of ongoing scientific investigation [62]. The results underscore the need for the careful monitoring of Al concentrations in EDs, particularly those packaged in aluminum cans, to ensure compliance with regulatory standards and minimize potential health risks.

As, Cd, and Pb are frequently found in the environment as pollutants. These metals have adverse effects on the human body and there is no known mechanism of homeostasis for them [63]. HMs are potentially toxic in high amounts or after long-term exposure. These metals manifest the ability to move and concentrate in different organs, leading to various health issues [19]. Therefore, monitoring their concentrations in various foods, including EDs, is warranted.

Comparing the concentrations of harmful HMs in the EDs to the available literature data, the following conclusions can be drawn: The studies by Kilic et al. [17], Adepoju and Ojo [64], and Bunu et al. [57] reported As levels ranging from 0.76 to 6.73 μg L^−1^, 2.1 to 7.1 μg L^−1^, and 0.5 to 60.3 μg L^−1^, respectively. The As levels in our study were significantly elevated in ED 7, with a statistic of 30.466 and an adjusted *p*-value of 0.005, which is below the 0.05 threshold for significance. This indicates that the As concentration in ED 7 surpasses the WHO, EU, and US EPA norms (Table 5), supporting the hypothesis that the As levels in this particular energy drink are higher than recommended. In addition, slightly increased levels of As were found in ED 8 and ED 9. Early symptoms of low As exposure in drinking water include melanosis (abnormal black-brown skin pigmentation) and keratosis (the hardening of the palms and soles). Ongoing exposure, on the other hand, leads to leukomelanosis (skin depigmentation with white patches), hyperkeratosis, and potentially skin cancer [65].

In previous research, Ni levels were reported to range between 0.93 and 22.7 μg L^−1^ [4], 12 and 59 μg L^−1^ [55], and 35.98 and 303.97 μg L^−1^ [17], significantly higher than our findings, which revealed Ni concentrations to be 3–46 times lower. Despite the relatively lower levels detected, the results of our risk assessment indicated that Ni posed no carcinogenic risk in adults, with the ILCR values falling below the safe threshold of 10^−6^ for most samples, except for ED 1 and ED 3, which presented a moderate risk. In adolescents, however, an intermediate cancer risk was identified across all the samples. Nickel exposure, depending on its concentration and duration, is associated with various adverse health effects, including contact dermatitis, cardiovascular diseases, asthma, lung fibrosis, and respiratory tract cancer [66].

In our investigation, the levels of Cd and Pb ranged from 0.19 to 0.78 μg L^−1^ and 5.00 to 32.79 μg L^−1^, respectively. It is important to mention that the levels of Pb in ED 3 exceeded the permitted levels for this metal in drinking water as stated in Table 5. Among the studies that were conducted, only the research by Adepoju and Ojo [64] reported significantly reduced levels of Cd (0.1–0.2 μg L^−1^) and Pb (1.6–4.8 μg L^−1^). In research conducted in Poland, Szymczycha-Madeja et al. [55] observed elevated levels of Cd (1.2–2.5 μg L^−1^) and Pb (19–53 μg L^−1^) compared to the findings of Leśniewicz et al. [4], who reported lower concentrations of Cd and Pb at 0.21–0.88 μg L^−1^ and 5.91–17.2 μg L^−1^, respectively. Our findings on the levels of these HMs were most similar to those of Leśniewicz et al. [4]. Kilic et al. [17] also reported comparable results for Cd and Pb. Moreover, Bunu et al. [57] found maximum Pb concentrations of 23.4 μg L^−1^ for Pb in their study. In the case of HM content in carbonated and non-carbonated beverages [59], the lowest mean concentration of Cd (7.4–18.6 μg L^−1^) followed by Pb (4.1–4.5 μg L^−1^) was observed in both types of beverage samples. A Pb range of 0.0014–6.0 μg L^−1^ was found in a prior investigation of fruit juices and non-alcoholic beverage samples from the USA [60]. Pb levels in a variety of soft drink samples packaged in glass bottles, plastic, and cans ranged from 0.5 to 6.44 μg L^−1^ [67]. According to earlier Egyptian research on soft drink samples, several soft drink brands had Cd concentrations ranging from 0.5 μg L^−1^ to 1.96 μg L^−1^ [61].

Researchers from Nigeria and Iraq observed particularly high levels of Cd and Pb concentrations [56,57,68,69,70] that were above the acceptable limits of these HMs in drinking water (Table 5). In the study conducted by Yahaya et al. [69], Cd levels ranged from 769 to 779 μg L^−1^ while Pb levels fell between 12 and 323 μg L^−1^. Mohammed and his colleagues [56] found Cd levels between 140 and 340 μg L^−1^ and Pb levels ranging from 100 to 140 μg L^−1^. Pb concentrations determined in the studies by Bunu et al. [68] and Gimba et al. [70] ranged from 18 to 332 μg L^−1^ and 28 to 139 μg L^−1^, respectively. Contamination with Pb is a significant issue in Nigeria. According to Momodu and Anyakora [71], 36.7% of the drinking water wells in Nigeria had Pb levels exceeding the WHO guideline value. Over a long period of intake, Cd can build up in the kidneys and liver, potentially causing kidney damage due to its extended biological half-life [72]. Excessive exposure to Pb can result in bone weakness, metallic taste, insomnia, seizures, microcytic anemia, glucosuria, cognitive issues, anorexia, reticulocytosis, and more. The organs affected are the brain, bone, blood, kidney, and thyroid [73].

The concentration levels of Cr found in this research (13.49–67.53 μg L^−1^), and also documented by Kilic et al. [17] (13.25–100.96 μg L^−1^) and Adepoju and Ojo [64] (534.3–608.9 μg L^−1^) are significantly greater than those reported in the ED samples in the Leśniewicz et al. [4] (2.56–19.7 μg L^−1^) and Szymczycha-Madeja et al. [55] (3.5–14 μg L^−1^) studies. The Cr concentrations exhibited significant deviations from the WHO and EU norms in two energy drinks: ED 9 and ED 3. ED 9 showed a moderate increase in the Cr levels (statistic = 10.746) with an adjusted *p*-value of 0.038, which is below the significance threshold of 0.05. ED 3 presented a substantial increase in the Cr concentration (statistic = 308.273) with an adjusted *p*-value of 0.000 indicating a highly significant departure from the norm. It is essential to maintain regular glucose metabolism in human nutrition by ensuring that Cr levels in drinking water are within the recommended range. However, excessive intake of Cr can result in stomach issues, ulcers, seizures, and damage to the kidneys and liver, possibly leading to death [74].

Our risk assessment revealed that the THQs for As, Pb, Cd, and Cr were below the threshold value of one, indicating no non-carcinogenic concerns for long-term exposure. However, the ILCR values in the adolescent age group highlighted notable carcinogenic risks for both As and Cr. Specifically, the ILCR values for As exceeded the safe threshold of 10^−6^ in seven samples, indicating intermediate cancer risk, while one sample surpassed the 10^−4^ level, signifying a high cancer risk. For Cr, the ILCR values exceeded 10^−6^ in seven samples, with two samples surpassing the 10^−4^ threshold, pointing to a high cancer risk. Importantly, our study measured the total Cr levels, encompassing both Cr^6+^ and Cr^3+^. While Cr^6+^ is recognized as a genotoxic and carcinogenic compound, Cr^3+^ is an essential trace element for human nutrition. This underscores the necessity for further speciation analysis to accurately assess the health risks associated with Cr exposure.

Our study found elevated levels of Mn, similar to the findings in the studies conducted by Kilic et al. [17] (5.45–489.93 μg L^−1^), Szymczycha-Madeja et al. [55] (9.4–586 μg L^−1^), and Mohammed et al. [56] (100–210 μg L^−1^). Leśniewicz et al. [4] discovered noticeably lower amounts of this element in ED samples (15.1–40.9 μg L^−1^). The Mn concentrations exceeded the EU and US EPA norms in two energy drinks: ED 5 and ED 8. ED 5 showed a significant increase in the Mn levels (statistic = 57.898) with an adjusted *p*-value of 0.001, while ED 8 also demonstrated elevated Mn concentrations (statistic = 37.589) with an adjusted *p*-value of 0.003. The carbonated and non-carbonated beverage samples examined by Ahmed et al. [59] showed mean Mn concentrations in the range 119.0–146.4 μg L^−1^, which was higher compared to our results. A previous report from Brazil also demonstrated the elevated mean content of Mn 150.4 μg L^−1^ in orange juices [75]. As widely recognized, Mn is a powerful neurotoxic in addition to being an essential element. Numerous enzymes involved in energy metabolism, the generation of neurotransmitters, the regulation of reproductive hormones, and endogenous antioxidant enzyme systems use it as a cofactor. On the other hand, excessive Mn exposure is hazardous, particularly to the central nervous system (CNS), since it gradually destroys nerve cells [76].

Except for Yahaya et al.’s research [69] reporting the highest Cu concentration of 14,041 μg L^−1^, other studies found Cu levels in EDs below drinking water standards (Table 5). The Cu concentrations in our study and in Martins et al. [49] did not exceed 20 μg L^−1^. However, Kilic et al. [17] observed Cu levels ranging from 23.67 to 60.48 μg L^−1^, Leśniewicz et al. [4] found concentrations between 0.87 and 109 μg L^−1^, and Szymczycha-Madeja et al. [55] recorded levels between 11 and 79 μg L^−1^. Juices and soft beverages’ Cu content was also examined in the literature. A comparison with our results showed that there are significantly lower Cu levels in EDs than in the aforementioned drinks. For example, in Brazil, orange fruit juices have been shown to contain Cu at concentrations between 21.6 and 293 μg L^−1^. According to Godebo et al. [60], orange juices have a Cu concentration of 75.15 to 141.66 μg L^−1^, which is higher than the concentration in another study [59]. Remarkably, a recent study on non-alcoholic juice found that two varieties had significantly higher mean concentrations of copper: 280.0 μg L^−1^ in fresh mango juice and 240.0 μg L^−1^ in cappy mango juice [61]. According to reports, the Cu concentration in samples of different soft drink brands in Egypt ranges from 10.0 to 66.64 μg L^−1^ [67]. Conversely, Abdel-Rahman et al. [61] found that the Cu concentrations in canned and plastic-bottled Pepsi drinks were 210.0 μg L^−1^ and 160.0 μg L^−1^, respectively. Cu acts as a cofactor for various enzymes (referred to as cuproenzymes) that play a role in energy production, iron metabolism, neuropeptide activation, connective tissue production, and neurotransmitter creation [70]. But, prolonged exposure to elevated copper levels can lead to liver damage and symptoms in the gastrointestinal tract such as abdominal pain, cramps, nausea, diarrhea, and vomiting [77].

Kilic et al. [17], Leśniewicz et al. [4], Bunu et al. [68], Yahaya et al. [69], and Mohammed et al. [56] all reported significantly high concentrations of Fe in their respective studies (334.77–937.12, 139–1380, 26–1850, 1083–1157, and 120–1030 μg L^−1^). In our research, the Fe levels varied between 121.86 and 308.31 μg L^−1^. All ED samples, with the exception of the EDs in the studies by Szymczycha-Madeja et al. [55] (35–78 μg L^−1^) and Martins et al. [49] (70–190 μg L^−1^), had higher Fe concentrations compared to the permitted levels (Table 5). Fe concentrations were generally within the EU and US EPA norms across most EDs. However, ED 8 exhibited a significant increase in Fe levels (statistic = 71.735) with an adjusted *p*-value of 0.001, indicating a substantial deviation from the norm. Fe is an important element in many biological processes, as on the one hand, it serves as an excellent oxygen carrier, and on the other, it can act as a protein-bound redox element [78]. Iron deficiency is common worldwide and in infants can cause severe neurological deficit. However, excessive iron intake is generally connected with an increased risk of colorectal cancer [79].

The analysis of Zn concentrations in our study, as well as in studies conducted by other researchers [4,49,55,57,68,69], revealed that Zn was present in different brands of EDs within acceptable limits (Table 5). The maximum Zn concentrations of 1005 μg L^−1^ and 2907 μg L^−1^ were observed by Yahaya et al. [69] and Bunu et al. [57], respectively. In turn, the Zn concentration in soft drinks packaged in cans, glass, and plastic bottles collected from Giza’s Egyptian market places was found to be in the range of 10 μg L^−1^ and 197.63 μg L^−1^ [67], which is less than the ranges obtained in both carbonated (265.15–315.78 μg L^−1^) and non-carbonated (168.81–301.81 μg L^−1^) beverage samples in the research study presented by Ahmed et al. [59]. Zinc plays a significant role in the metabolism of proteins, lipids, nucleic acids, and gene transcription [80]. Its function in the human body is wide-ranging in terms of reproduction, immune function, and wound healing. Zinc deficiency may manifest as growth issues, sexual problems, inflammation, digestive symptoms, or skin problems [81].

The present study and other available scientific reports have shown that EDs are not inert to human health. Nevertheless, there is no document in the EU legal system that specifies what an “energy drink” is. Regulation (EU) No 1169/2011 on the provision of food information to consumers mainly refers to a non-alcoholic caffeine-rich soft drink. From 2014 onwards, drinks containing more than 15 mg caffeine/100 mL had to be labeled with the warning: ‘High caffeine content. Not recommended for serving to children, pregnant and breastfeeding women’ and information on the caffeine content in mg/100 mL [82]. In Denmark, it is illegal to produce or distribute soft drinks and to add more than 15 mg of caffeine per 100 milliliters to food. EDs are only allowed to be sold to people above 16 in the UK and Germany, but only to people above 18 in Lithuania and Latvia [83]. Energy drinks are categorized as dietary supplements or ordinary foods in the US. The Dietary Supplement Health and Education Act of 1994 (DSHEA) states that substances used in normal foods, including caffeine, cannot be used in dietary supplements without previous FDA approval. Despite caffeine’s classification as a medication, the DSHEA does not mandate that components in herbal supplements be disclosed [84]. Since 1 January 2024, Poland has prohibited the sale of energy drinks to anyone younger than 18. With the exception of natural sources, it covers items that contain more than 150 mg of taurine or caffeine per liter. This rule attempts to shield youth from health consequences that can impair everyday functioning and education, such as sleeplessness, anxiety, or difficulty focusing. These rules were put in place with the goal of encouraging a healthy lifestyle and helping children develop good eating habits at a young age.

## 5. Conclusions

The analysis of nine ED samples revealed the presence of Na, K, Mg, and Ca, with Na being the most abundant. These macro-elements are crucial for the overall health of the body. Overall, the majority of the elements analyzed in the EDs adhered to the WHO, EU, and US EPA norms, with no significant deviations observed. However, notable exceptions included elevated levels of Cr (in ED 3 and ED 9), Mn (in ED 5 and ED 8), Fe (in ED 8), As (in ED 7), Sr (in ED 3), and Al (in ED 6, ED 3 and ED 8). The observed differences in heavy metal content may be due to their heterogeneity, raw material sources, origin, production, and packaging processes. Consequently, caution is advised when consuming EDs, as excessive drinking can lead to increased build-up of both essential and non-essential TEs and/or HMs in the human body. In general, the accumulation of these metals can increase the risk of non-communicable diseases such as cardiovascular diseases and various types of cancer. These findings raise specific concerns regarding the safety of certain EDs concerning the Cr, Mn, Fe, As, Sr, and Al content, warranting further investigation and potential regulatory scrutiny to ensure consumer safety.

The analysis of nine ED samples revealed the presence of Na, K, Mg, and Ca, with Na being the most abundant. These macro-elements are essential for maintaining overall health. Most of the elements analyzed adhered to the WHO, EU, and US EPA norms, with no significant deviations observed for non-carcinogenic risks, as indicated by the THQ values below one for all the elements, including Cr, Mn, Fe, As, Sr, and Al. This confirms that in terms of long-term non-carcinogenic exposure, the investigated EDs are generally safe for consumption.

However, carcinogenic risk assessments highlighted concerns, particularly for certain elements like As and Cr in some ED samples, especially in the adolescent age group. The ILCR values for these elements exceeded the thresholds for moderate to high carcinogenic risk in specific cases, necessitating caution. The variability in heavy metal content among the ED samples may be attributed to differences in raw material sources, production processes, and packaging.

While no immediate non-carcinogenic risks were identified, excessive and prolonged consumption of EDs could contribute to the accumulation of both essential and non-essential trace elements and/or heavy metals in the human body, posing potential long-term health risks, including carcinogenic risks. These findings underscore the importance of regulatory oversight and further studies, including longitudinal and clinical research, to ensure the safety of energy drink consumption and protect consumer health.

## Figures and Tables

**Table 1:** Composition of energy drink samples investigated in our study.

Energy Drink	Composition
ED 1	water, sugar, citric acid, carbon dioxide, taurine (400 mg/100 mL), acidity regulator (sodium citrates), glucose–fructose syrup, caffeine (30 mg/100 mL), flavors, colors (ammonia caramel and riboflavin), sweeteners (acesulfame K and sucralose), inositol (20 mg/100 mL), and vitamins (niacin, pantothenic acid, vitamin B6, and vitamin B12)
ED 2	water, sugar, carbon dioxide, acids (citric acid and tartaric acid), acidity regulator (sodium citrates), flavors, taurine (100 mg/100 mL), preservatives (potassium sorbate and sodium benzoate), caffeine (30 mg/100 mL), vitamins (niacin and vitamin B6), sweetener (sucralose), inositol, and colors (E102, E129, and E133)
ED 3	water, sucrose, glucose, acid (citric acid), carbon dioxide, taurine (400 mg/100 mL), acidity regulator (sodium carbonates and magnesium carbonate), caffeine (30 mg/100 mL), vitamins (niacin, pantothenic acid, B6, and B12), flavors, and colors (caramel and riboflavin)
ED 4	water, sugar, acidity regulators (citric acid and sodium citrates), carbon dioxide, taurine (350 mg/100 mL), flavor, caffeine (30 mg/100 mL), dyes (E 150d), riboflavin, and vitamins (niacin, pantothenic acid, vitamin B6, and vitamin B12)
ED 5	water, apple juice from concentrated apple juice, sugar, carbon dioxide, acid (citric acid), taurine (400 mg/100 mL), flavor, caffeine (30 mg/100 mL), sweeteners (acesulfame K and aspartame), color (E 150d), and vitamins (niacin, vitamin B6, and vitamin B12)
ED 6	water, sugar, acid (citric acid), carbon dioxide, taurine (300 mg/100 mL), acidity regulator (sodium citrates), flavor, caffeine (30 mg/100 mL), safflower color concentrate, inositol, and vitamins (niacin, pantothenic acid, vitamin B6, and vitamin B12)
ED 7	water, acid (citric acid), carbon dioxide, taurine (400 mg/100 mL), acidity regulator (sodium citrate), flavors, ginseng extract (0.08%), sweeteners (sucralose and acesulfame K), caffeine (30 mg/100 mL), preservatives (sorbic acid and benzoic acid), L-carnitine tartrate (0.04%), vitamins (niacin, pantothenic acid, vitamin B6, and vitamin B12), sodium chloride, D-glucuronolactone, guarana seed extract (0.002%), and inositol
ED 8	water, apple juice from concentrated juice (10%), grape juice from concentrated juice (10%), acidity regulators (citric acid, sodium citrates), carbon dioxide, sweeteners (sucralose and acesulfame K), caffeine (30 mg/100 mL), inositol, colors (ammonia caramel, sulfite caramel, and riboflavin), flavors, flavor enhancer (erythritol), and vitamins (niacin, B6, B12, and pantothenic acid)
ED 9	water, sugar, carbon dioxide, magnesium citrate, citric acid, flavorings, rhubarb gum, glycerol and vegetable resin esters, caffeine (30 mg/100 mL), and vitamins (niacin, vitamin B6, vitamin B12, and biotin)

**Table 2:** ICP-OES instrument operating conditions.

RF power [W]	1000
Plasma flow [L min^−1^]	15
Auxiliary flow [L min^−1^]	1.5
Nebulizer flow [L min^−1^]	0.75
Replicate read time [s]	1
Instrument stabilization delay [s]	15
Sample uptake delay [s]	15
Pump rate [rpm]	15
Rinse time [s]	10
Wavelength [nm]	Ca 396.847, K 766.491, Mg 279.533, Na 589.592

**Table 3:** ICP-MS instrument operating conditions.

RF power [W]	1600
Plasma flow [L min^−1^]	15
Carrier gas flow rate [L min^−1^]	0.34
Dilution gas flow rate [L min^−1^]	0.57
Nebulizer	MicroMist
Ion Lenses	x-Lens
Replicates	3
Stabilization time [s]	30
Acquisition Mode	Spectrum
Nebulizer pump speed [rpm]	0.1
Energy discrimination [V]	5
Isotopes acquired	^11^B, ^27^Al, ^51^V, ^52^Cr, ^55^Mn, ^56^Fe, ^59^Co, ^60^Ni, ^63^Cu, ^66^Zn, ^75^As, ^88^Sr, ^111^Cd, ^137^Ba, ^208^Pb

**Table 4:** Content of the macro-minerals [mg L^−1^] in the analyzed energy drinks (*n* = 27).

Elements	Min	Max	Mean	SD	p.adj *
Sodium (Na)	8.62	619.85	377.16	223.25	0.00524
Potassium (K)	5.51	189.57	65.28	72.90	0.00552
Magnesium (Mg)	0.013	543.97	83.96	184.14	0.00476
Calcium (Ca)	2.54	116.1	23.15	35.66	0.00692

* *p.adj* —*p*-values after Bonferroni correction

**Table 5:** Content of micro-minerals in the analyzed energy drinks (*n* = 27) and the WHO, EU, and US EPA regulations of maximum permissible metal levels in drinking water [µg L^−1^].

Elements	Min	Max	Mean	SD	p.adj *	WHO [42]	EU [43]	US EPA [44,45,46]
Boron (B)	194.01	796.82	432.28	186.54	1.00000	2400	1500	1400
Aluminum (Al)	227.54	456.97	297.32	67.18	0.43800	N/A	200	200
Vanadium (V)	0.29	10.44	3.60	3.04	0.02640	N/A	N/A	N/A **
Chromium (Cr)	13.49	67.53	39.70	18.26	0.02520	50	50	100
Manganese (Mn)	6.69	107.27	27.38	35.27	0.05415	N/A	50	50
Iron (Fe)	121.86	308.31	205.34	66.00	0.22950	300	200	300
Cobalt (Co)	0.19	4.13	1.20	1.18	0.01830	N/A	N/A	100
Nickel (Ni)	2.04	6.57	3.36	1.57	0.14745	70	20	100
Copper (Cu)	2.94	16.76	8.02	4.31	0.02985	2000	2000	1300
Zinc (Zn)	10.34	64.56	23.98	17.27	0.03960	N/A	N/A	5000
Arsenic (As)	1.57	23.05	7.33	7.00	0.02625	10	10	10
Strontium (Sr)	2.99	3878.21	452.74	1284.72	0.02400	N/A	N/A	1500
Cadmium (Cd)	0.19	0.78	0.34	0.18	1.00000	3	5	5
Barium (Ba)	4.81	19.98	11.54	5.18	0.04095	1300	N/A	2000
Lead (Pb)	5.00	32.79	10.37	8.53	1.00000	10	10	15

* *p.adj* —*p*-values after Bonferroni correction; N/A—not available; ** a proposed notification level for vanadium at 15 μg L^−1^ in drinking water established by the OEHHA [47].

**Table 6:** Estimated daily intake and health risks associated with micro-mineral exposure in adolescents from energy drink consumption.

Sample	EDI THQ															HI
	B	Al	V	Cr	Mn	Fe	Co	Ni	Cu	Zn	As	Sr	Cd	Ba	Pb	
ED 1	1.6 × 10^−3^ 8.1 × 10^−3^	1.5 × 10^−3^ 1.5 × 10^−3^	3.4 × 10^−5^ 3.7 × 10^−3^	1.2 × 10^−4^ 3.9 × 10^−2^	4.0 × 10^−5^ 2.9 × 10^−4^	5.8 × 10^−4^ 8.3 × 10^−4^	4.4 × 10^−6^ 1.5 × 10^−2^	1.8 × 10^−5^ 9.0 × 10^−4^	5.6 × 10^−5^ 1.4 × 10^−3^	2.2 × 10^−4^ 7.2 × 10^−4^	5.3 × 10^−6^ 1.8 × 10^−2^	1.3 × 10^−4^ 2.1 × 10^−4^	2.6 × 10^−6^ 5.2 × 10^−3^	4.8 × 10^−5^ 6.8 × 10^−4^	2.7 × 10^−5^ 7.7 × 10^−3^	0.10
ED 2	6.7 × 10^−4^ 3.4 × 10^−3^	7.7 × 10^−4^ 7.7 × 10^−4^	6.7 × 10^−6^ 7.5 × 10^−4^	9.4 × 10^−5^ 3.1 × 10^−2^	2.4 × 10^−5^ 1.7 × 10^−4^	4.1 × 10^−4^ 5.9 × 10^−4^	6.4 × 10^−7^ 2.1 × 10^−3^	9.4 × 10^−6^ 4.7 × 10^−4^	2.4 × 10^−5^ 6.1 × 10^−4^	6.7 × 10^−5^ 2.2 × 10^−4^	1.4 × 10^−5^ 4.7 × 10^−2^	1.1 × 10^−5^ 1.8 × 10^−5^	1.4 × 10^−6^ 2.8 × 10^−3^	2.2 × 10^−5^ 3.1 × 10^−4^	2.8 × 10^−5^ 8.1 × 10^−3^	0.10
ED 3	6.4 × 10^−4^ 3.2 × 10^−3^	1.0 × 10^−3^ 1.0 × 10^−3^	1.7 × 10^−5^ 1.9 × 10^−3^	2.1 × 10^−4^ 6.9 × 10^−2^	2.4 × 10^−5^ 1.7 × 10^−4^	5.1 × 10^−4^ 7.3 × 10^−4^	1.4 × 10^−5^ 4.6 × 10^−2^	2.2 × 10^−5^ 1.1 × 10^−3^	3.6 × 10^−5^ 8.9 × 10^−4^	3.5 × 10^−5^ 1.2 × 10^−4^	1.4 × 10^−5^ 4.6 × 10^−2^	1.3 × 10^−2^ 2.2 × 10^−2^	9.1 × 10^−7^ 1.8 × 10^−3^	6.7 × 10^−5^ 9.6 × 10^−4^	1.1 × 10^−4^ 3.1 × 10^−2^	0.23
ED 4	1.9 × 10^−3^ 9.7 × 10^−3^	9.4 × 10^−4^ 9.4 × 10^−4^	1.0 × 10^−5^ 1.1 × 10^−3^	1.9 × 10^−4^ 6.5 × 10^−2^	2.4 × 10^−5^ 1.7 × 10^−4^	6.7 × 10^−4^ 9.6 × 10^−4^	1.6 × 10^−6^ 5.5 × 10^−3^	7.3 × 10^−6^ 3.6 × 10^−4^	2.2 × 10^−5^ 5.5 × 10^−4^	5.1 × 10^−5^ 1.7 × 10^−4^	8.5 × 10^−6^ 2.8 × 10^−2^	8.0 × 10^−5^ 1.3 × 10^−4^	1.0 × 10^−6^ 2.0 × 10^−3^	2.6 × 10^−5^ 3.8 × 10^−4^	2.2 × 10^−5^ 6.2 × 10^−3^	0.12
ED 5	1.6 × 10^−3^ 8.1 × 10^−3^	7.7 × 10^−4^ 7.7 × 10^−4^	1.7 × 10^−5^ 1.9 × 10^−3^	1.2 × 10^−4^ 4.0 × 10^−2^	2.2 × 10^−4^ 1.6 × 10^−3^	7.2 × 10^−4^ 1.0 × 10^−3^	3.5 × 10^−6^ 1.2 × 10^−2^	1.0 × 10^−5^ 5.0 × 10^−4^	1.8 × 10^−5^ 4.4 × 10^−4^	9.7 × 10^−5^ 3.2 × 10^−4^	1.1 × 10^−5^ 3.8 × 10^−2^	7.7 × 10^−5^ 1.3 × 10^−4^	1.1 × 10^−6^ 2.3 × 10^−3^	5.1 × 10^−5^ 7.3 × 10^−4^	3.2 × 10^−5^ 9.2 × 10^−3^	0.12
ED 6	1.4 × 10^−3^ 6.9 × 10^−3^	1.0 × 10^−3^ 1.0 × 10^−3^	1.0 × 10^−5^ 1.1 × 10^−3^	1.0 × 10^−4^ 3.5 × 10^−2^	8.1 × 10^−5^ 5.8 × 10^−4^	8.7 × 10^−4^ 1.2 × 10^−3^	1.3 × 10^−6^ 4.3 × 10^−3^	7.6 × 10^−6^ 3.8 × 10^−4^	2.4 × 10^−5^ 6.1 × 10^−4^	6.6 × 10^−5^ 2.2 × 10^−4^	1.3 × 10^−5^ 4.2 × 10^−2^	6.1 × 10^−5^ 1.0 × 10^−4^	6.4 × 10^−7^ 1.3 × 10^−3^	4.2 × 10^−5^ 6.0 × 10^−4^	3.1 × 10^−5^ 8.9 × 10^−3^	0.10
ED 7	2.7 × 10^−3^ 1.3 × 10^−2^	1.0 × 10^−3^ 1.0 × 10^−3^	1.0 × 10^−5^ 1.1 × 10^−3^	4.7 × 10^−5^ 1.6 × 10^−2^	4.0 × 10^−5^ 2.9 × 10^−4^	1.0 × 10^−3^ 1.5 × 10^−3^	4.7 × 10^−6^ 1.6 × 10^−2^	8.5 × 10^−6^ 4.3 × 10^−4^	1.3 × 10^−5^ 3.4 × 10^−4^	3.8 × 10^−5^ 1.3 × 10^−4^	7.7 × 10^−5^ 2.6 × 10^−1^	1.0 × 10^−5^ 1.7 × 10^−5^	6.4 × 10^−7^ 1.3 × 10^−3^	1.6 × 10^−5^ 2.3 × 10^−4^	2.5 × 10^−5^ 7.0 × 10^−3^	0.32
ED 8	1.3 × 10^−3^ 6.7 × 10^−3^	9.1 × 10^−4^ 9.1 × 10^−4^	1.7 × 10^−6^ 1.9 × 10^−4^	9.4 × 10^−5^ 3.1 × 10^−2^	3.7 × 10^−4^ 2.6 × 10^−3^	9.5 × 10^−4^ 1.4 × 10^−3^	3.9 × 10^−6^ 1.3 × 10^−2^	1.2 × 10^−5^ 5.9 × 10^−4^	3.9 × 10^−5^ 9.7 × 10^−4^	1.2 × 10^−4^ 3.8 × 10^−4^	3.6 × 10^−5^ 1.2 × 10^−1^	2.4 × 10^−4^ 4.1 × 10^−4^	1.2 × 10^−6^ 2.4 × 10^−3^	5.3 × 10^−5^ 7.5 × 10^−4^	2.2 × 10^−5^ 6.2 × 10^−3^	0.19
ED 9	1.2 × 10^−3^ 5.9 × 10^−3^	9.7 × 10^−4^ 9.7 × 10^−4^	1.0 × 10^−6^ 1.1 × 10^−4^	2.3 × 10^−4^ 7.6 × 10^−2^	2.4 × 10^−5^ 1.7 × 10^−4^	4.6 × 10^−4^ 6.6 × 10^−4^	2.5 × 10^−6^ 8.4 × 10^−3^	6.9 × 10^−6^ 3.4 × 10^−4^	9.9 × 10^−6^ 2.5 × 10^−4^	3.9 × 10^−5^ 1.3 × 10^−4^	4.2 × 10^−5^ 1.4 × 10^−1^	5.1 × 10^−5^ 8.5 × 10^−5^	8.7 × 10^−7^ 1.7 × 10^−3^	2.4 × 10^−5^ 3.4 × 10^−4^	1.7 × 10^−5^ 4.8 × 10^−3^	0.24

**Table 7:** Estimated daily intake and health risks associated with micro-mineral exposure in adults from energy drink consumption.

Sample	EDI THQ															HI
	B	Al	V	Cr	Mn	Fe	Co	Ni	Cu	Zn	As	Sr	Cd	Ba	Pb	
ED 1	6.8 × 10^−4^ 3.4 × 10^−3^	6.5 × 10^−4^ 6.5 × 10^−4^	1.4 × 10^−5^ 1.6 × 10^−3^	4.9 × 10^−5^ 1.6 × 10^−2^	1.7 × 10^−5^ 1.2 × 10^−4^	2.4 × 10^−4^ 3.5 × 10^−4^	1.8 × 10^−6^ 6.1 × 10^−3^	7.6 × 10^−6^ 3.8 × 10^−4^	2.4 × 10^−5^ 5.9 × 10^−4^	9.1 × 10^−5^ 3.0 × 10^−4^	2.2 × 10^−6^ 7.4 × 10^−3^	5.3 × 10^−5^ 8.9 × 10^−5^	1.1 × 10^−6^ 2.2 × 10^−3^	2.0 × 10^−5^ 2.9 × 10^−4^	1.1 × 10^−5^ 3.2 × 10^−3^	0.04
ED 2	2.8 × 10^−4^ 1.4 × 10^−3^	3.2 × 10^−4^ 3.2 × 10^−4^	2.8 × 10^−6^ 3.1 × 10^−4^	3.9 × 10^−5^ 1.3 × 10^−2^	9.9 × 10^−6^ 7.0 × 10^−5^	1.7 × 10^−4^ 2.5 × 10^−4^	2.7 × 10^−7^ 8.9 × 10^−4^	3.9 × 10^−6^ 2.0 × 10^−4^	1.0 × 10^−5^ 2.6 × 10^−4^	2.8 × 10^−5^ 9.4 × 10^−5^	5.9 × 10^−6^ 2.0 × 10^−2^	4.4 × 10^−6^ 7.4 × 10^−6^	5.9 × 10^−7^ 1.2 × 10^−3^	9.1 × 10^−6^ 1.3 × 10^−4^	1.2 × 10^−5^ 3.4 × 10^−3^	0.04
ED 3	2.7 × 10^−4^ 1.3 × 10^−3^	4.4 × 10^−4^ 4.4 × 10^−4^	7.0 × 10^−6^ 7.8 × 10^−4^	8.7 × 10^−5^ 2.9 × 10^−2^	9.9 × 10^−6^ 7.0 × 10^−5^	2.1 × 10^−4^ 3.1 × 10^−4^	5.8 × 10^−6^ 1.9 × 10^−2^	9.3 × 10^−6^ 4.6 × 10^−4^	1.5 × 10^−5^ 3.7 × 10^−4^	1.5 × 10^−5^ 4.9 × 10^−5^	5.8 × 10^−6^ 1.9 × 10^−2^	5.5 × 10^−3^ 9.1 × 10^−3^	3.8 × 10^−7^ 7.6 × 10^−4^	2.8 × 10^−5^ 4.0 × 10^−4^	4.6 × 10^−5^ 1.3 × 10^−2^	0.10
ED 4	8.2 × 10^−4^ 4.1 × 10^−3^	3.9 × 10^−4^ 3.9 × 10^−4^	4.2 × 10^−6^ 4.7 × 10^−4^	8.2 × 10^−5^ 2.7 × 10^−2^	9.9 × 10^−6^ 7.0 × 10^−5^	2.8 × 10^−4^ 4.0 × 10^−4^	6.9 × 10^−7^ 2.3 × 10^−3^	3.1 × 10^−6^ 1.5 × 10^−4^	9.2 × 10^−6^ 2.3 × 10^−4^	2.1 × 10^−5^ 7.1 × 10^−5^	3.6 × 10^−6^ 1.2 × 10^−2^	3.4 × 10^−5^ 5.6 × 10^−5^	4.2 × 10^−7^ 8.5 × 10^−4^	1.1 × 10^−5^ 1.6 × 10^−4^	9.1 × 10^−6^ 2.6 × 10^−3^	0.05
ED 5	6.8 × 10^−4^ 3.4 × 10^−3^	3.2 × 10^−4^ 3.2 × 10^−4^	7.0 × 10^−6^ 7.8 × 10^−4^	5.1 × 10^−5^ 1.7 × 10^−2^	9.2 × 10^−5^ 6.5 × 10^−4^	3.0 × 10^−4^ 4.3 × 10^−4^	1.5 × 10^−6^ 4.9 × 10^−3^	4.2 × 10^−6^ 2.1 × 10^−4^	7.4 × 10^−6^ 1.8 × 10^−4^	4.1 × 10^−5^ 1.4 × 10^−4^	4.7 × 10^−6^ 1.6 × 10^−2^	3.2 × 10^−5^ 5.4 × 10^−5^	4.8 × 10^−7^ 9.6 × 10^−4^	2.1 × 10^−5^ 3.0 × 10^−4^	1.4 × 10^−5^ 3.9 × 10^−3^	0.05
ED 6	5.8 × 10^−4^ 2.9 × 10^−3^	4.2 × 10^−4^ 4.2 × 10^−4^	4.2 × 10^−6^ 4.7 × 10^−4^	4.4 × 10^−5^ 1.5 × 10^−2^	3.4 × 10^−5^ 2.4 × 10^−4^	3.6 × 10^−4^ 5.2 × 10^−4^	5.4 × 10^−7^ 1.8 × 10^−3^	3.2 × 10^−6^ 1.6 × 10^−4^	1.0 × 10^−5^ 2.6 × 10^−4^	2.8 × 10^−5^ 9.2 × 10^−5^	5.3 × 10^−6^ 1.8 × 10^−2^	2.6 × 10^−5^ 4.3 × 10^−5^	2.7 × 10^−7^ 5.4 × 10^−4^	1.8 × 10^−5^ 2.5 × 10^−4^	1.3 × 10^−5^ 3.7 × 10^−3^	0.04
ED 7	1.1 × 10^−3^ 5.6 × 10^−3^	4.2 × 10^−4^ 4.2 × 10^−4^	4.2 × 10^−6^ 4.7 × 10^−4^	2.0 × 10^−5^ 6.6 × 10^−3^	1.7 × 10^−5^ 1.2 × 10^−4^	4.3 × 10^−4^ 6.2 × 10^−4^	2.0 × 10^−6^ 6.6 × 10^−3^	3.6 × 10^−6^ 1.8 × 10^−4^	5.7 × 10^−6^ 1.4 × 10^−4^	1.6 × 10^−5^ 5.4 × 10^−5^	3.2 × 10^−5^ 1.1 × 10^−1^	4.2 × 10^−6^ 7.0 × 10^−6^	2.7 × 10^−7^ 5.4 × 10^−4^	6.8 × 10^−6^ 9.7 × 10^−5^	1.0 × 10^−5^ 3.0 × 10^−3^	0.13
ED 8	5.6 × 10^−4^ 2.8 × 10^−3^	3.8 × 10^−4^ 3.8 × 10^−4^	7.0 × 10^−7^ 7.8 × 10^−5^	3.9 × 10^−5^ 1.3 × 10^−2^	1.5 × 10^−4^ 1.1 × 10^−3^	4.0 × 10^−4^ 5.7 × 10^−4^	1.6 × 10^−6^ 5.4 × 10^−3^	5.0 × 10^−6^ 2.5 × 10^−4^	1.6 × 10^−5^ 4.1 × 10^−4^	4.8 × 10^−5^ 1.6 × 10^−4^	1.5 × 10^−5^ 5.1 × 10^−2^	1.0 × 10^−4^ 1.7 × 10^−4^	4.9 × 10^−7^ 9.9 × 10^−4^	2.2 × 10^−5^ 3.2 × 10^−4^	9.0 × 10^−6^ 2.6 × 10^−3^	0.08
ED 9	4.9 × 10^−4^ 2.5 × 10^−3^	4.1 × 10^−4^ 4.1 × 10^−4^	4.2 × 10^−7^ 4.7 × 10^−5^	9.6 × 10^−5^ 3.2 × 10^−2^	9.9 × 10^−6^ 7.0 × 10^−5^	1.9 × 10^−4^ 2.8 × 10^−4^	1.1 × 10^−6^ 3.5 × 10^−3^	2.9 × 10^−6^ 1.4 × 10^−4^	4.1 × 10^−6^ 1.0 × 10^−4^	1.6 × 10^−5^ 5.4 × 10^−5^	1.8 × 10^−5^ 5.9 × 10^−2^	2.1 × 10^−5^ 3.6 × 10^−5^	3.7 × 10^−7^ 7.3 × 10^−4^	9.9 × 10^−6^ 1.4 × 10^−4^	7.0 × 10^−6^ 2.0 × 10^−3^	0.10

**Table 8:** Incremental lifetime cancer risk (ILCR) for adolescents and adults due to Cr, Ni, As, Cd, and Pb exposure from the analyzed energy drinks.

Sample	ILCR *				
	Cr	Ni	As	Cd	Pb
Adolescents					
ED 1	5.9 × 10^−5^	3.1 × 10^−5^	7.9 × 10^−6^	1.0 × 10^−6^	2.3 × 10^−7^
ED 2	4.7 × 10^−5^	1.6 × 10^−5^	2.1 × 10^−5^	5.4 × 10^−7^	2.4 × 10^−7^
ED 3	1.0 × 10^−4^	3.8 × 10^−5^	2.1 × 10^−5^	3.4 × 10^−7^	9.4 × 10^−7^
ED 4	9.7 × 10^−5^	1.2 × 10^−5^	1.3 × 10^−5^	3.8 × 10^−7^	1.8 × 10^−7^
ED 5	6.0 × 10^−5^	1.7 × 10^−5^	1.7 × 10^−5^	4.3 × 10^−7^	2.7 × 10^−7^
ED 6	5.2 × 10^−5^	1.3 × 10^−5^	1.9 × 10^−5^	2.4 × 10^−7^	2.6 × 10^−7^
ED 7	2.4 × 10^−5^	1.4 × 10^−5^	1.2 × 10^−4^	2.4 × 10^−7^	2.1 × 10^−7^
ED 8	4.7 × 10^−5^	2.0 × 10^−5^	5.4 × 10^−5^	4.5 × 10^−7^	1.8 × 10^−7^
ED 9	1.1 × 10^−4^	1.2 × 10^−5^	6.3 × 10^−5^	3.3 × 10^−7^	1.4 × 10^−7^
Adults					
ED 1	2.5 × 10^−5^	1.3 × 10^−5^	3.3 × 10^−6^	4.2 × 10^−7^	9.7 × 10^−8^
ED 2	2.0 × 10^−5^	6.7 × 10^−6^	8.9 × 10^−6^	2.2 × 10^−7^	1.0 × 10^−7^
ED 3	4.4 × 10^−5^	1.6 × 10^−5^	8.7 × 10^−6^	1.4 × 10^−7^	3.9 × 10^−7^
ED 4	4.1 × 10^−5^	5.2 × 10^−6^	5.3 × 10^−6^	1.6 × 10^−7^	7.7 × 10^−8^
ED 5	2.5 × 10^−5^	7.2 × 10^−6^	7.1 × 10^−6^	1.8 × 10^−7^	1.2 × 10^−7^
ED 6	2.2 × 10^−5^	5.4 × 10^−6^	7.9 × 10^−6^	1.0 × 10^−7^	1.1 × 10^−7^
ED 7	9.9 × 10^−6^	6.1 × 10^−6^	4.9 × 10^−5^	1.0 × 10^−7^	8.8 × 10^−8^
ED 8	2.0 × 10^−5^	8.5 × 10^−6^	2.3 × 10^−5^	1.9 × 10^−7^	7.7 × 10^−8^
ED 9	4.8 × 10^−5^	4.9 × 10^−6^	2.7 × 10^−5^	1.4 × 10^−7^	6.0 × 10^−8^

* ILCR was calculated by multiplying the EDI by the cancer slope factor (CSF). Oral CSF for Cr, Ni, inorganic As, Cd, and Pb is 0.5, 1.7, 1.5, 0.38, and 0.0085 mg/kg/day, respectively [48].

## Data Availability

Data are contained within the article.

## References

[1] Costantino A., Maiese A., Lazzari J., Casula C., Turillazzi E., Frati P., Fineschi V. (2023). The Dark Side of Energy Drinks: A Comprehensive Review of Their Impact on the Human Body. Nutrients.

[2] Erdmann J., Wiciński M., Wódkiewicz E., Nowaczewska M., Słupski M., Otto S.W., Kubiak K., Huk-Wieliczuk E., Malinowski B. (2021). Effects of Energy Drink Consumption on Physical Performance and Potential Danger of Inordinate Usage. Nutrients.

[3] Hladun O., Papaseit E., Martín S., Barriocanal A.M., Poyatos L., Farré M., Pérez-Mañá C. (2021). Interaction of Energy Drinks with Prescription Medication and Drugs of Abuse. Pharmaceutics.

[4] Leśniewicz A., Grzesiak M., Żyrnicki W., Borkowska-Burnecka J. (2016). Mineral Composition and Nutritive Value of Isotonic and Energy Drinks. Biol. Trace Elem. Res..

[5] Nowak D., Jasionowski A. (2015). Analysis of the Consumption of Caffeinated Energy Drinks among Polish Adolescents. Int. J. Environ. Res. Public Health.

[6] Gutiérrez-Hellín J., Varillas-Delgado D. (2021). Energy Drinks and Sports Performance, Cardiovascular Risk, and Genetic Associations; Future Prospects. Nutrients.

[7] Jagim A.R., Harty P.S., Barakat A.R., Erickson J.L., Carvalho V., Khurelbaatar C., Camic C.L., Kerksick C.M. (2022). Prevalence and Amounts of Common Ingredients Found in Energy Drinks and Shots. Nutrients.

[8] Kumar G., Park S., Onufrak S. (2015). Perceptions about Energy Drinks Are Associated with Energy Drink Intake among U.S. Youth. Am. J. Health Promot..

[9] Dobmeyer D.J., Stine R.A., Leier C.V., Greenberg R., Schaal S.F. (1983). The Arrhythmogenic Effects of Caffeine in Human Beings. N. Engl. J. Med..

[10] Tomanic M., Paunovic K., Lackovic M., Djurdjevic K., Nestorovic M., Jakovljevic A., Markovic M. (2022). Energy Drinks and Sleep among Adolescents. Nutrients.

[11] Ehlers A., Marakis G., Lampen A., Hirsch-Ernst K.I. (2019). Risk Assessment of Energy Drinks with Focus on Cardiovascular Parameters and Energy Drink Consumption in Europe. Food Chem. Toxicol..

[12] Fletcher E.A., Lacey C.S., Aaron M., Kolasa M., Occiano A., Shah S.A. (2017). Randomized Controlled Trial of High-volume Energy Drink versus Caffeine Consumption on ECG and Hemodynamic Parameters. J. Am. Heart Assoc..

[13] Higgins J.P., Liras G.N., Liras I.N., Jacob R., Husain F., Pabba K.C., Schultea M. (2021). Energy Drink Effects on Hemodynamics and Endothelial Function in Young Adults. Cardiology.

[14] Shah S.A., Szeto A.H., Farewell R., Shek A., Fan D., Quach K.N., Bhattacharyya M., Elmiari J., Chan W., O’Dell K. (2019). Impact of High Volume Energy Drink Consumption on Electrocardiographic and Blood Pressure Parameters: A Randomized Trial. J. Am. Heart Assoc..

[15] Trapp G.S., Hurworth M., Jacoby P., Maddison K., Allen K., Martin K., Christian H., Ambrosini G.L., Oddy W., Eastwood P.R. (2021). Energy Drink Intake Is Associated with Insomnia and Decreased Daytime Functioning in Young Adult Females. Public Health Nutr..

[16] Trapp G.S.A., Allen K., O’Sullivan T.A., Robinson M., Jacoby P., Oddy W.H. (2014). Energy Drink Consumption Is Associated with Anxiety in Australian Young Adult Males. Depress. Anxiety.

[17] Kilic S., Cengiz M.F., Kilic M. (2018). Monitoring of Metallic Contaminants in Energy Drinks Using ICP-MS. Environ. Monit. Assess..

[18] Eticha T., Hymete A. (2014). Health Risk Assessment of Heavy Metals in Locally Produced Beer to the Population in Ethiopia. J. Bioanal. Biomed..

[19] Scutarașu E.C., Trincă L.C. (2023). Heavy Metals in Foods and Beverages: Global Situation, Health Risks and Reduction Methods. Foods.

[20] Shaheen N., Irfan N.M., Khan I.N., Islam S., Islam M.S., Ahmed M.K. (2016). Presence of Heavy Metals in Fruits and Vegetables: Health Risk Implications in Bangladesh. Chemosphere.

[21] Izah S.C., Inyang I.R., Angaye T.C.N., Okowa I.P. (2016). A Review of Heavy Metal Concentration and Potential Health Implications of Beverages Consumed in Nigeria. Toxics.

[22] Charehsaz M., Helvacıoğlu S., Çetinkaya S., Demir R., Erdem O., Aydin A. (2021). Heavy Metal and Essential Elements in Beers from Turkey Market: A Risk Assessment Study. Hum. Exp. Toxicol..

[23] Helvacıoğlu S., Charehsaz M., Gulhane O.E., Aydın A. (2021). Assessment of Toxic Element Content of Some Grape Molasses Produced by Conventional and Industrial Techniques: Insights into Human Safety. Toxin Rev..

[24] DesMarais T.L., Costa M. (2019). Mechanisms of Chromium-Induced Toxicity. Curr. Opin. Toxicol..

[25] Pourret O., Hursthouse A. (2019). It’s Time to Replace the Term “Heavy Metals” with “Potentially Toxic Elements” When Reporting Environmental Research. Int. J. Environ. Res. Public Health.

[26] Witkowska D., Słowik J., Chilicka K. (2021). Heavy Metals and Human Health: Possible Exposure Pathways and the Competition for Protein Binding Sites. Molecules.

[27] El Hosry L., Sok N., Richa R., Al Mashtoub L., Cayot P., Bou-Maroun E. (2023). Sample Preparation and Analytical Techniques in the Determination of Trace Elements in Food: A Review. Foods.

[28] Ali H., Khan E., Ilahi I. (2019). Environmental Chemistry and Ecotoxicology of Hazardous Heavy Metals: Environmental Persistence, Toxicity, and Bioaccumulation. J. Chem..

[29] Cannas D., Loi E., Serra M., Firinu D., Valera P., Zavattari P. (2020). Relevance of Essential Trace Elements in Nutrition and Drinking Water for Human Health and Autoimmune Disease Risk. Nutrients.

[30] Czarnek K., Tatarczak-Michalewska M., Szopa A., Klimek-Szczykutowicz M., Jafernik K., Majerek D., Blicharska E. (2024). Bioaccumulation Capacity of Onion (*Allium cepa* L.) Tested with Heavy Metals in Biofortification. Molecules.

[31] Nduka J.K., Kelle H.I., Amuka J.O. (2019). Health Risk Assessment of Cadmium, Chromium and Nickel from Car Paint Dust from Used Automobiles at Auto-Panel Workshops in Nigeria. Toxicol. Rep..

[32] Eze V., Ndife C., Muogbo M. (2021). Carcinogenic and Non-Carcinogenic Health Risk Assessment of Heavy Metals in Njaba River, Imo State, Nigeria. Braz. J. Anal. Chem..

[33] EFSA Gathering Consumption Data on Specific Consumer Groups of Energy Drinks. https://www.efsa.europa.eu/en/supporting/pub/en-394.

[34] US EPA Human Health Risk Assessment. https://semspub.epa.gov/work/03/2339323.pdf.

[35] Montgomery D.C. (2017). The Kruskal-Wallis Test. Design and Analysis of Experiments.

[36] Liu H. (2015). Comparing Welch’s ANOVA, a Kruskal-Wallis Test and Traditional ANOVA in Case of Heterogeneity of Variance. Master Thesis.

[37] Hochberg Y. (1988). A Sharper Bonferroni Procedure for Multiple Tests of Significance. Biometrika.

[38] Fay M.P., Proschan M.A. (2010). Wilcoxon-Mann-Whitney or t-Test? on Assumptions for Hypothesis Tests and Multiple Interpretations of Decision Rules. Stat. Surv..

[39] R Core Team (2024). R: A Language and Environment for Statistical Computing.

[40] Wickham H., Averick M., Bryan J., Chang W., McGowan L., François R., Grolemund G., Hayes A., Henry L., Hester J. (2019). Welcome to the Tidyverse. J. Open Source Softw..

[41] Kassambara A. Rstatix: Pipe-Friendly Framework for Basic Statistical Tests 2023. https://cran.r-project.org/web/packages/rstatix/index.html.

[42] World Health Organization (2017). Guidelines for Drinking-Water Quality—Fourth Edition Incorporating the First Addendum.

[43] European Commission Directive (EU) 2020/2184 of the European Parliament and of the Council of 16 December 2020 on the Quality of Water Intended for Human Consumption (Recast) (Text with EEA Relevance) 2020. https://eur-lex.europa.eu/eli/dir/2020/2184/oj.

[44] US EPA Announcement of Preliminary Regulatory Determinations for Contaminants on the Third Drinking Water Contaminant Candidate List. U.S. Environmental Protection Agency Web. https://www.federalregister.gov/documents/2014/10/20/2014-24582/announcement-of-preliminary-regulatory-determinations-forcontaminants-on-the-third-drinking-water.

[45] US EPA National Primary Drinking Water Regulations 2008. https://www.epa.gov/ground-water-and-drinking-water/national-primary-drinking-water-regulations.

[46] US EPA Secondary Drinking Water Regulations: Guidance for Nuisance Chemicals 2013. https://www.epa.gov/sdwa/secondary-drinking-water-standards-guidance-nuisance-chemicals.

[47] OEHHA Proposed Notification Level for Vanadium. California Office of Environmental Health Hazard Assessment (OEHHA). https://oehha.ca.gov/water/notification-level/proposed-notification-level-vanadium.

[48] US EPA Risk Assessment Guidance for Superfund Volume I: Human Health Evaluation Manual (Part E, Supplemental Guidance for Dermal Risk Assessment). 2004, EPA/540/R/99/005. https://www.epa.gov/sites/default/files/2015-09/documents/part_e_final_revision_10-03-07.pdf.

[49] Martins A.S., Junior J.B.P., De Araújo Gomes A., Carvalho F.I.M., Filho H.A.D., Das Graças Fernandes Dantas K. (2020). Mineral Composition Evaluation in Energy Drinks Using ICP OES and Chemometric Tools. Biol. Trace Elem. Res..

[50] Calliope S.R., Samman N.C. (2020). Sodium Content in Commonly Consumed Foods and Its Contribution to the Daily Intake. Nutrients.

[51] Nurmilah S., Cahyana Y., Utama G.L., Aït-Kaddour A. (2022). Strategies to Reduce Salt Content and Its Effect on Food Characteristics and Acceptance: A Review. Foods.

[52] Salman E., Kadota A., Miura K. (2024). Global Guidelines Recommendations for Dietary Sodium and Potassium Intake. Hypertens. Res..

[53] (2012). World Health Organization Guideline: Sodium Intake for Adults and Children.

[54] World Health Organization Tackling NCDs: “best Buys” and Other Recommended Interventions for the Prevention and Control of Noncommunicable Diseases 2017. https://iris.who.int/bitstream/handle/10665/259232/WHO-NMH-NVI-17.9-eng.pdf?sequence=1.

[55] Szymczycha-Madeja A., Welna M., Pohl P. (2013). Determination of Elements in Energy Drinks by ICP OES with Minimal Sample Preparation. J. Braz. Chem. Soc..

[56] Mohammed S.G., Al-Hashimi A.G., Al-Hussainy K.S. (2012). Determination of Caffeine and Trace Minerals Contents in Soft and Energy Drinks Available in Basrah Markets. Pak. J. Nutr..

[57] Bunu S.J., Ebeshi B.U., Kpun H.F., Kashimawo A.J., Vaikosen E.N., Itodo C.B. (2023). Atomic Absorption Spectroscopic (AAS) Analysis of Heavy Metals and Health Risks Assessment of Some Common Energy Drinks. Pharmacol. Toxicol. Nat. Med..

[58] Francisco B.B.A., Brum D.M., Cassella R.J. (2015). Determination of Metals in Soft Drinks Packed in Different Materials by ETAAS. Food Chem..

[59] Ahmed M., Yousaf A., Khaleeq A., Saddiqa A., Sanaullah M., Ahmad W., Ali I., Khalid K., Wani T.A., Zargar S. (2024). Chemometric Analysis and Human Health Implications of Trace and Heavy/Non-Essential Metals through Ingestion of Carbonated and Non-Carbonated Beverages. Biol. Trace Elem. Res..

[60] Godebo T.R., Stoner H., Pechilis M., Taylor-Arnold H., Ashmead J., Claman L., Guest L., Consolati W., DiMatteo O., Johnson M. (2023). Toxic Metals and Essential Elements Contents in Commercially Available Fruit Juices and Other Non-Alcoholic Beverages from the United States. J. Food Compos. Anal..

[61] Abdel-Rahman G.N., Ahmed M.B.M., Sabry B.A., Ali S.S.M. (2019). Heavy Metals Content in Some Non-Alcoholic Beverages (Carbonated Drinks, Flavored Yogurt Drinks, and Juice Drinks) of the Egyptian Markets. Toxicol. Rep..

[62] WHO Aluminium in Drinking-Water. Background Document for Preparation of WHO Guidelines for Drinking-Water Quality 2010. https://cdn.who.int/media/docs/default-source/wash-documents/wash-chemicals/aluminium.pdf?sfvrsn=e54f4db9_4.

[63] Llobet J.M., Falcó G., Casas C., Teixidó A., Domingo J.L. (2003). Concentrations of Arsenic, Cadmium, Mercury, and Lead in Common Foods and Estimated Daily Intake by Children, Adolescents, Adults, and Seniors of Catalonia, Spain. J. Agric. Food Chem..

[64] Adepoju O.T., Ojo V.O. (2014). Consumption Pattern of Energy Drinks by University of Ibadan Students and Associated Health Risks Factors. FNS.

[65] Dastgiri S., Mosaferi M., Fizi M.A.H., Olfati N., Zolali S., Pouladi N., Azarfam P. (2010). Arsenic Exposure, Dermatological Lesions, Hypertension, and Chromosomal Abnormalities among People in a Rural Community of Northwest Iran. J. Health Popul. Nutr..

[66] Genchi G., Carocci A., Lauria G., Sinicropi M.S., Catalano A. (2020). Nickel: Human Health and Environmental Toxicology. Int. J. Environ. Res. Public. Health.

[67] Ghuniem M.M., Khorshed M.A., El-Safty S.M., Souaya E.R., Khalil M.M. (2022). Potential Human Health Risk Assessment of Potentially Toxicelements Intake via Consumption of Soft Drinks Purchasedfrom Different Egyptian Markets. Int. J. Environ. Anal. Chem..

[68] Bunu S.J., George D., Alfred-Ugbenbo D., Ebeshi B.U. (2023). Heavy Metals Quantification and Correlative Carcinogenic Risks Evaluation in Selected Energy Drinks Sold in Bayelsa State Using Atomic Absorption Spectroscopic Technique. Int. J. Chem. Res..

[69] Yahaya T.O., Gulumbe B.H., Umar A.K., Yusuf A., Mohammed A.Z., Izuafa A., Abubakar A. (2022). Heavy Metal Content and Associated Health Risks in Selected Energy Drinks Sold in Birnin Kebbi, Nigeria. AJHSE.

[70] Gimba C.E., Abechi S.E., Abbas N.S. (2014). Studies on Physicochemical Properties, Trace Mineral and Heavy Metal Contents of Common Energy Drinks. Int. J. Adv. Res..

[71] Momodu M.A., Anyakora C.A. (2010). Heavy Metal Contamination of Ground Water: The Surulere Case Study. Res. J. Environ. Earth Sci..

[72] Rafati Rahimzadeh M., Rafati Rahimzadeh M., Kazemi S., Moghadamnia A. (2017). Cadmium Toxicity and Treatment: An Update. Casp. J. Intern. Med..

[73] Sachdeva C., Thakur K., Sharma A., Sharma K.K. (2018). Lead: Tiny but Mighty Poison. Indian. J. Clin. Biochem..

[74] Khan S.A., Uddin Z., Zubair A. (2011). Levels of Selected Heavy Metals in Drinking Water of Peshawar City. Int. J. Sci. Nat..

[75] De Souza M.J., Barciela-Alonso M.C., Aboal-Somoza M., Bermejo-Barrera P. (2021). Determination of the Trace Element Contents of Fruit Juice Samples by ICP OES and ICP-MS. Braz. J. Anal. Chem..

[76] Gunter T.E., Miller L.M., Gavin C.E., Eliseev R., Salter J., Buntinas L., Alexandrov A., Hammond S., Gunter K.K. (2004). Determination of the Oxidation States of Manganese in Brain, Liver, and Heart Mitochondria. J. Neurochem..

[77] National Research Council (US) (2000). Copper in Drinking Water. Committee on Copper in Drinking Water.

[78] Huang X. (2003). Iron Overload and Its Association with Cancer Risk in Humans: Evidence for Iron as a Carcinogenic Metal. Mutat. Res..

[79] Ashmore J.H., Rogers C.J., Kelleher S.L., Lesko S.M., Hartman T.J. (2016). Dietary Iron and Colorectal Cancer Risk: A Review of Human Population Studies. Crit. Rev. Food Sci. Nutr..

[80] McClung J.P. (2019). Iron, Zinc, and Physical Performance. Biol. Trace Elem. Res..

[81] Sanna A., Firinu D., Zavattari P., Valera P. (2018). Zinc Status and Autoimmunity: A Systematic Review and Meta-Analysis. Nutrients.

[82] Binder J.-H., Gortsos C.V. (2015). The European Banking Union: A Compendium.

[83] Schroeder W. (2016). Age Restrictions on the Sale of Energy Drinks from an EU Law Perspective. Eur. Food Feed. Law Rev. (EFFL).

[84] Generali J.A. (2013). Energy Drinks: Food, Dietary Supplement, or Drug?. Hosp. Pharm..

